# Epitalon and Epithalamin: A Narrative Review of Mechanistic, Preclinical, and Clinical Evidence

**DOI:** 10.3390/ijms27198922

**Published:** 2026-10-08

**Authors:** Ankur Jain, David M. Gallacher, Mohammed A. Issa, Timothy R. Deer, Robert J. Yong, Sudhir Diwan, Woojin Lee, Dawood Sayed, Kasra Amirdelfan, Christopher L. Robinson

**Affiliations:** 1 Department of Physical Medicine and Rehabilitation, The Johns Hopkins University School of Medicine, Baltimore, MD 21205, USA; ajain67@jh.edu; 2 Department of Anesthesiology, Perioperative and Pain Medicine, Brigham and Women’s Hospital, Harvard Medical School, Boston, MA 02115, USA; 3 Spine and Nerve Center of the Virginias, West Virginia University, Thomas Health Campus, Charleston, WV 25309, USA; 4 Albert Einstein College of Medicine, Bronx, NY 10461, USA; 5 Department of Anesthesiology, Warren Alpert Medical School, Brown University, Providence, RI 02912, USA; 6 Department of Anesthesiology, The University of Kansas School of Medicine, Kansas City, MO 66160, USA; 7 Boomerang Healthcare, Walnut Creek, CA 94598, USA; 8 Division of Pain, Department of Anesthesiology and Critical Care Medicine, The Johns Hopkins University School of Medicine, Baltimore, MD 21205, USA

**Keywords:** epitalon, AEDG, epithalamin, telomeres, aging, regenerative medicine, circadian rhythm, peptide therapeutics, longevity

## Abstract

Epitalon (Ala-Glu-Asp-Gly) is a synthetic tetrapeptide developed from the amino-acid composition of epithalamin, a chemically distinct pineal-derived polypeptide preparation. This narrative review evaluates the mechanistic, preclinical, and human evidence for synthetic epitalon in the context of regenerative and longevity medicine, while considering epithalamin studies separately as historical context. PubMed and Google Scholar were searched through 22 September 2026, with primary experimental and clinical studies prioritized and evidence appraised by study design, model, sample size, intervention, outcomes, safety reporting, and independent replication. Epitalon has been reported to modulate telomere-maintenance pathways, circadian and neuroendocrine function, oxidative stress, and cellular aging markers. Preclinical studies suggest biological activity, but direct evidence in musculoskeletal disease models is lacking. Human evidence is limited to small physiological or biomarker studies and an older controlled study in retinitis pigmentosa; no adequately powered randomized trial has established therapeutic efficacy. Pharmacokinetic, dose-ranging, and long-term safety data remain inadequate, while oncologic, immunogenicity, product-quality, and regulatory uncertainties following a 2026 FDA advisory review remain unresolved. Epitalon is therefore biologically active but clinically unproven, and further standardized pharmacologic, safety, and disease-specific studies are needed before clinical translation.

## 1. Introduction

Aging is a major risk factor for many chronic diseases, including cardiovascular disease, cancer, diabetes, and neurodegenerative disorders [1]. These conditions develop through a range of age-related cellular changes. Telomere shortening, mitochondrial dysfunction, cellular senescence, and chronic inflammation are among the processes most consistently implicated [2].

The musculoskeletal system is also strongly affected by aging. Osteoarthritis, osteoporosis, sarcopenia, and degenerative tendinopathy all become more common with age [3]. These disorders share several features of biological aging, including oxidative stress, inflammation, cellular senescence, and a reduced capacity for tissue repair [2,3,4]. Current management is largely symptomatic, aimed at improving pain and function rather than reversing the underlying degenerative process. This has increased interest in biological approaches that may modify tissue degeneration or promote repair. Several regenerative medicine therapies have gained attention, including platelet-rich plasma (PRP), bone marrow aspirate concentrate (BMAC), and investigational peptide-based approaches [5,6,7,8,9].

As interest in peptide-based therapies increases within the field of regenerative medicine, several compounds have received considerable attention. Body Protection Compound-157 (BPC-157) is one of them, with preclinical studies reporting tissue-repair effects, although it remains unapproved by the U.S. Food and Drug Administration (FDA) and is subject to regulatory restrictions [9,10]. Epitalon differs from BPC-157 in that most of its published work has focused on biological aging, particularly telomere biology and neuroendocrine regulation, rather than direct musculoskeletal tissue repair [11,12]. The epitalon literature remains limited and is concentrated within a small number of research groups.

Epitalon is a synthetic tetrapeptide (Ala-Glu-Asp-Gly) based on the amino acid composition of epithalamin, a polypeptide extract from the bovine pineal gland [11,12,13]. Although epitalon was developed from the amino-acid composition of epithalamin, the two preparations are chemically distinct and their experimental and clinical evidence should not be considered interchangeable.

Epitalon was developed and extensively investigated by Khavinson and colleagues at the St. Petersburg Institute of Bioregulation and Gerontology [12,13]. Direct studies of epitalon have reported effects involving telomerase activity and neuroendocrine regulation, whereas much of the earlier antioxidant and human longevity literature was generated using epithalamin and is considered separately in this review.

Telomerase contributes to the maintenance of telomere length at chromosome ends and supports chromosomal stability [14,15]. BPC-157 has been studied mainly in relation to wound-healing and tissue-repair pathways [9], whereas epitalon research has centered more on telomere biology and neuroendocrine regulation [11,12]. Telomere shortening has also been associated with osteoarthritis in observational and meta-analytic studies [16,17]. These associations do not establish that epitalon itself has therapeutic effects in musculoskeletal disease.

Independent replication remains limited. A 2025 study reported human telomerase reverse transcriptase (hTERT) upregulation and telomere elongation in several human cell models [11]. A subsequent correction replaced several figures, so the reported telomerase-activity findings should be interpreted cautiously [11]. The correction specifically states that the first three figures of the original Al-Dulaimi et al. publication were incor-rect and were replaced [11].

In July 2026, the FDA Pharmacy Compounding Advisory Committee (PCAC) reviewed epitalon free base and epitalon acetate for possible inclusion on Section 503A Bulk Drug Substances List for insomnia [18]. Although FDA’s briefing materials recommended against inclusion, the committee subsequently recommended inclusion by a narrow favorable vote. That recommendation is advisory and does not represent FDA approval or establish safety or efficacy.

Araj et al. published a comprehensive review of epitalon’s biological activity, experimental findings, and physicochemical properties in 2025 [12]. Since that review was published, new evidence has emerged. An independent study reported epitalon-associated telomere elongation in both normal and malignant human cell models, although the subsequent correction requires caution when interpreting some of the telomerase findings [11]. FDA also evaluated epitalon-related bulk drug substances through its 2026 PCAC process [18]. The present review incorporates these developments and critically examines the available evidence, with particular attention to the distinction between synthetic epitalon and epithalamin, limitations of human studies, including potentially overlapping cohorts, and unresolved safety concerns. We also examine the potential relevance of epitalon to regenerative and musculoskeletal medicine and identify the evidence needed before clinical use can be supported.

## 2. Literature Search and Review Approach

The literature search for this narrative review was conducted using PubMed and Google Scholar on 26 August 2026, and updated on 22 September 2026. The updated PubMed search used a predefined search strategy encompassing epitalon, epithalamin, and alternative names and identified 233 records. Three Google Scholar searches were conducted using predefined queries. Results were screened in relevance order, with screening limited to the first five pages per query. No publication-date or language restrictions were applied. The complete search strategies are provided in Supplementary Table S1. As this review was conducted as a narrative review, formal record-level counts for screening, exclusion, and final inclusion were not prospectively tracked. Records were evaluated for relevance and eligibility during evidence selection. A retrospective audit of the final evidence base identified 31 unique primary research publications directly investigating epitalon and/or epithalamin. Of these, 28 evaluated epitalon and 5 evaluated epithalamin, including two publications that investigated both preparations. Among the 28 epitalon publications, 4 involved administration to human participants and 24 were preclinical, mechanistic, or analytical studies.

Primary experimental and clinical studies directly investigating synthetic epitalon or epithalamin were prioritized. Relevant mechanistic studies, regulatory assessments, and selected reviews were also considered for biological and translational context. Publications unrelated to either preparation or the review’s central biological questions were excluded. Studies involving other peptides were considered when relevant to the comparative discussion. Duplicate records were counted once, and potentially overlapping clinical cohorts were identified to avoid treating repeated reports as independent evidence.

Two authors independently reviewed relevant full-text studies, with disagreements resolved through discussion and consensus. Evidence was critically appraised according to study design, experimental model, sample size, intervention characteristics, comparator groups, outcomes, statistical reporting, safety reporting, and independent replication, with study-level characteristics and limitations summarized in Table 1, Table 2, Table 3 and Table 4. Studies involving synthetic epitalon were evaluated separately from those involving epithalamin. Given the heterogeneous evidence base and narrative scope of this review, no formal risk-of-bias tool was applied, and no meta-analysis was performed. Study limitations were instead evaluated using a structured qualitative approach.

## 3. Preparation and Pharmacology

Epitalon has a molecular weight of approximately 390.35 Da [13]. It was developed as a synthetic tetrapeptide based on the amino-acid composition of epithalamin, but it is not chemically identical to the parent preparation. The two preparations should therefore not be considered interchangeable. Epithalamin is a heterogeneous pineal-derived polypeptide preparation, whereas epitalon consists of the defined tetrapeptide Ala-Glu-Asp-Gly (AEDG) [13]. Consequently, findings obtained with epithalamin are treated in this review as historical or mechanistic context unless directly reproduced with synthetic epitalon. FDA likewise distinguishes the two substances rather than treating the names as interchangeable. In its 2026 Pharmacy Compounding Advisory Committee evaluation, FDA separately considered epitalon free base and epitalon acetate as related but distinct bulk drug substances [18].

Epitalon contains three asymmetric centers and therefore has eight possible stereoisomeric configurations; the form described in the experimental literature is composed of L-amino acids [12]. Its conformational properties have also been investigated directly using molecular-dynamics modeling. A 2006 study simulated zwitterionic epitalon in aqueous solution for 1500 ps using an AMBER molecular-mechanics force field and found that its structure can be stabilized by two intramolecular salt bridges between the N-terminal nitrogen and carboxylate oxygen atoms of the Asp and Glu side chains, together with an intramolecular hydrogen bond [19]. The authors noted that these interactions restrict, but do not eliminate, conformational flexibility. The biological significance of these modeled conformations remains uncertain [19].

Published studies provide limited detail regarding standardized pharmaceutical manufacture and formulation of epitalon. FDA’s 2026 evaluation similarly concluded that epitalon free base and epitalon acetate were not well characterized from a physical and chemical standpoint [18]. FDA also noted that the free-base and acetate forms are distinct chemical entities that may differ in physicochemical, pharmacokinetic, and pharmacodynamic properties.

Epitalon has been administered by several routes across the preclinical and human literature, including subcutaneous, intramuscular, intranasal, oral, sublingual, and parabulbar administration [20,21,22,23,24,25]. In the lifespan study in mice, epitalon was administered subcutaneously at a dose of 1.0 µg per mouse, approximately 30–40 µg/kg, for 5 consecutive days each month. Treatment began when the mice were 3 months old and continued until their natural death [21]. Intranasal administration was also studied, with findings suggesting it can modulate pineal secretory activity under stress conditions [22].

In aged rats, oral epitalon administration for one month altered regional glucose transport in the small intestine and increased glycine absorption in the proximal intestinal segment [26]. These findings demonstrate biological effects following oral administration but do not establish systemic bioavailability of intact epitalon. Human studies have used substantially different intramuscular, sublingual, and parabulbar regimens [23,24,25]. This heterogeneity in species, route, dose, and treatment schedule limits direct comparison across studies and prevents reliable determination of an optimal human dosing regimen.

Analytical characterization has also been reported for products marketed as epitalon outside authorized pharmaceutical channels. One study identified AEDG in two such preparations using liquid chromatography and mass spectrometry [27]. The analysis confirmed AEDG identity but did not evaluate pharmaceutical-grade purity, dosing accuracy, sterility, stability, or product safety [27]. Confirmation of molecular identity alone should therefore not be considered equivalent to pharmaceutical-quality characterization.

A major pharmacological limitation is the lack of adequate pharmacokinetic characterization. FDA identified one preliminary study in pregnant rabbits in which fluorescently labeled epitalon was detected in maternal and fetal tissues one hour after a single intravenous dose. However, the study did not establish that the detected fluorescence specifically represented intact labeled epitalon [18]. FDA identified no additional nonclinical pharmacokinetic or toxicokinetic studies of epitalon free base or epitalon acetate and found no human pharmacokinetic data [18]. Systemic bioavailability, metabolic fate, elimination, terminal half-life, and exposure-response relationships therefore remain undefined [18].

By comparison, BPC-157 has preliminary pharmacokinetic characterization in rats and dogs assessing intravenous and intramuscular exposure, elimination half-life, bioavailability, distribution, metabolism, and excretion [28]. These findings cannot be extrapolated to epitalon but illustrate the type of pharmacological characterization currently lacking [28]. For epitalon, the absence of pharmacokinetic data represents a significant barrier to dose selection and clinical translation. Future studies should therefore prioritize formal pharmacokinetic and pharmacodynamic characterization together with dose-ranging studies before broader clinical investigation.

## 4. Mechanisms of Action in Tissue Protection and Repair

### 4.1. Telomerase Activation and Telomere Biology

One of the most extensively studied proposed mechanisms of epitalon is the activation of telomerase, a ribonucleoprotein that contributes to the maintenance of telomere length at the ends of chromosomes [14,15]. Telomeres generally shorten with successive divisions in somatic cells, contributing to replicative aging. Once telomeres reach a critically short length, cells may enter replicative senescence, a state in which cells permanently stop dividing and begin releasing inflammatory signals that can contribute to tissue degeneration [29,30].

Khavinson et al. reported that epitalon induced telomerase activity and telomere elongation in human fetal fibroblast cell cultures [31]. The original study reported induction of expression of the telomerase catalytic subunit, enzymatic telomerase activity, and telomere elongation following epitalon exposure. In a subsequent study, epitalon treatment of aging human fibroblasts was associated with telomere elongation and continued proliferative capacity beyond the division limit observed in untreated control cells [32].

Khavinson et al. also evaluated AEDG in cultured blood lymphocytes from 11 healthy male donors [33]. Relative telomere length increased in five donors, decreased in two, and remained unchanged in four. Mitotic activity was largely unaffected. These findings suggest that AEDG may influence telomere length in a cell- and donor-dependent manner, although the small sample and variable responses limit interpretation [33].

A 2025 independent study by Al-Dulaimi et al. reported increased hTERT messenger RNA (mRNA) expression following epitalon exposure in several normal and malignant human cell lines [11]. The subsequently corrected dataset showed different telomere-maintenance responses between normal and malignant cells. Telomerase activity increased significantly in normal IBR.3 fibroblasts and human mammary epithelial cells after three weeks of treatment but did not increase significantly in the 21NT or BT474 breast-cancer cell lines. Telomere length increased in all four cell models under the treatment conditions studied, whereas alternative lengthening of telomeres (ALT) activity increased primarily in the malignant cell lines [11]. The corrected article explicitly distinguishes the telomerase response of the normal cells from the ALT-dominant response in the cancer lines. These results suggest that epitalon can influence telomere-maintenance pathways through mechanisms that differ by cell type.

**Figure 1:**
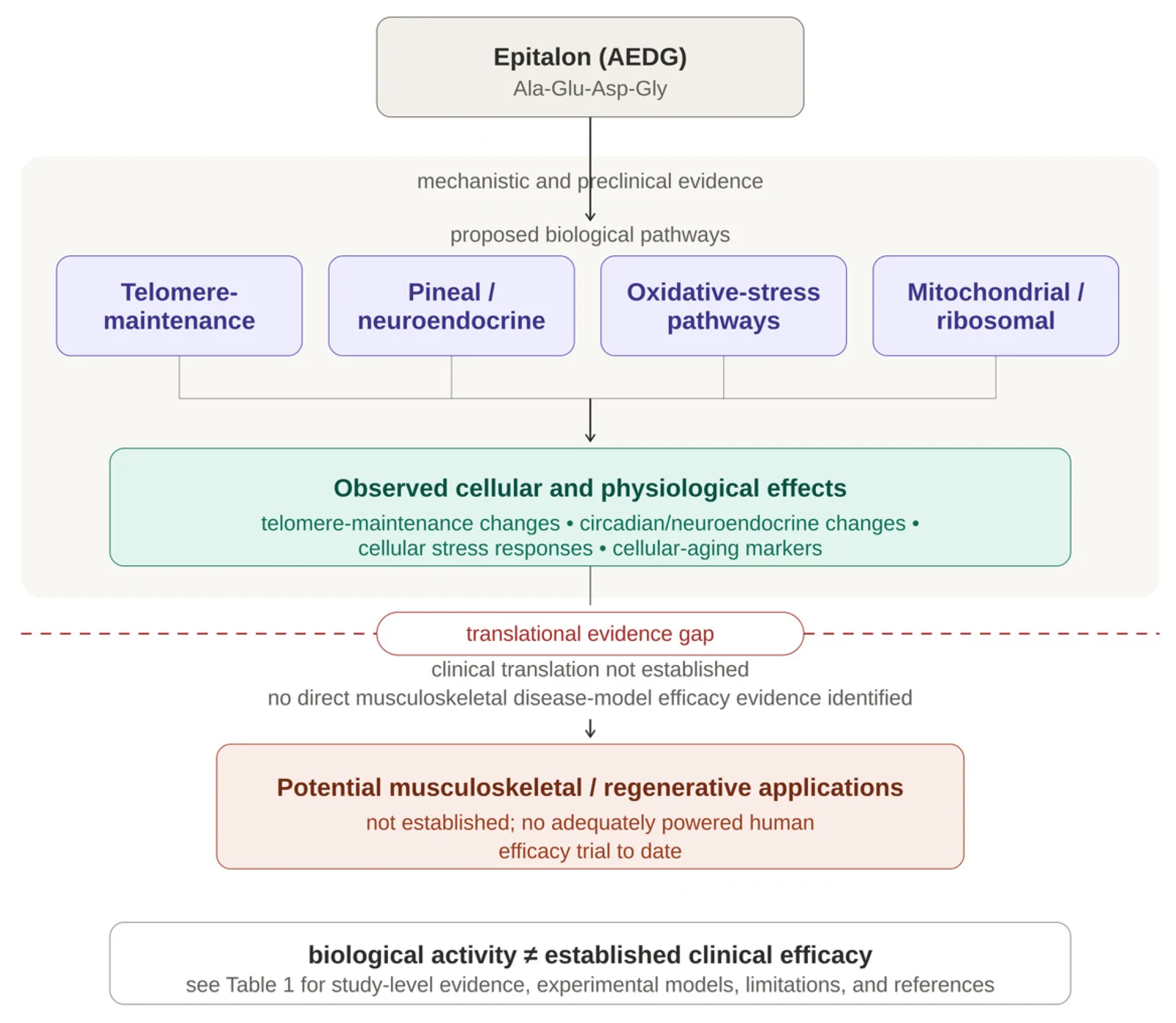
Conceptual framework for the proposed biological actions and translational evidence gap of synthetic epitalon (AEDG). Mechanistic and preclinical studies have reported effects involving telomere-maintenance pathways, pineal and neuroendocrine regulation, oxidative-stress pathways, and mitochondrial/ribosomal processes. These findings are associated with cellular and physiological changes but have not established therapeutic efficacy for musculoskeletal disease or regenerative-medicine applications. No direct musculoskeletal disease-model efficacy evidence was identified in the literature reviewed. Potential musculoskeletal and regenerative applications therefore remain investigational. Study-level evidence, experimental models, limitations, and references are summarized in Table 1; Abbreviation: AEDG, Ala-Glu-Asp-Gly.

However, these findings remain cellular and mechanistic and should not be interpreted as evidence that epitalon-mediated telomere elongation improves clinical outcomes or extends human lifespan. In addition, the potential oncological implications of telomerase activation and ALT require separate consideration, as discussed below in the safety and translational sections. The proposed mechanisms of epitalon are summarized in Figure 1, while the supporting evidence and its principal limitations are outlined in Table 1.

**Table 1:** Selected mechanistic evidence and limitations for synthetic epitalon (AEDG).

Proposed Mechanism	Model/Setting	n/Experimental Details	Key Findings	Main Limitation	Reference
Telomerase activation/telomere maintenance	Human fibroblasts and other human cell lines, in vitro; blood lymphocytes, ex vivo	Lymphocyte study: n = 11 healthy male donors. 2025 study: 21NT, BT474, IBR.3, and HMEC models; cancer cells treated for 4 days and normal cells for 3 weeks.	↑ hTERT mRNA in multiple cell models; ↑ telomere length in normal and malignant cells; telomerase activity ↑ in IBR.3 and HMEC but not significantly in 21NT or BT474; ALT ↑ mainly in malignant cells.	Cellular evidence with limited independent replication; clinical benefit remains unproven.	[11,31,32,33]
Melatonin/circadian regulation	Female rhesus monkeys, in vivo; young and old Wistar rat pineal glands, in vitro	2001 primate study: 6 young + 6 senescent monkeys, with 3 animals per age/treatment group. 2005 study: 17 young + 17 old monkeys. Rat study: 24 young + 12 old rats.	↑ evening melatonin and normalized cortisol rhythm in senescent monkeys; broader endocrine effects in aged primates; no significant direct effect on basal or isoproterenol-stimulated melatonin secretion in isolated rat pineal glands.	Animal evidence; small primate groups in the 2001 study and context-dependent effects.	[20,34,35]
Antioxidant/cellular stress response	Aged-animal studies; high-glucose ARPE-19 retinal pigment epithelial cells, in vitro	ARPE-19 cells exposed to 35 mM glucose and treated with epitalon at 20, 40, or 60 ng/mL. Conventional sample n was not clearly reported for the older antioxidant study.	Antioxidant effects reported; in ARPE-19 cells, ↓ ROS, improved scratch-wound closure, and reduced EMT- and fibrosis-related changes.	Older antioxidant evidence partly involves epithalamin; newer evidence is limited to an in vitro retinal model.	[36,37]
Mitochondrial/ribosomal regulation	Human pineal-gland cell cultures undergoing replicative aging, in vitro	Young cultures at passage 3 and aged cultures at passage 14; AEDG 100 ng/mL added at each subculture; 10 visual fields analyzed per case.	↑ MitoTracker Red staining area ~1.5-fold and ↓ L7A expression ~22% in aged pineal-cell cultures.	Single in vitro study without independent replication; systemic relevance remains unknown.	[38]

**Abbreviations:** AEDG, Ala-Glu-Asp-Gly; ALT, alternative lengthening of telomeres; EMT, epithelial–mesenchymal transition; HMEC, human mammary epithelial cells; hTERT, human telomerase reverse transcriptase; ROS, reactive oxygen species; ↑, increase; ↓, decrease. **Note:** Sample size is reported where clearly available from the primary study; otherwise, the relevant experimental details are provided.

Telomere biology and its relationship with musculoskeletal degeneration have been studied in the recent literature. A meta-analysis of six case–control studies found that patients with osteoarthritis had significantly shorter leukocyte telomere length compared to healthy controls [16]. Data from the Osteoarthritis Initiative cohort demonstrated that accelerated telomere loss is an independent risk factor for knee osteoarthritis (*p* = 0.041) [17]. A 2024 study also stated that moderate pain and severe pain in patients with osteoarthritis (OA) were inversely associated with leukocyte telomere length [39]. This finding aligns with evidence linking telomere shortening and cellular senescence with pro-inflammatory signaling that may contribute to joint degeneration [4].

These associations do not establish that epitalon itself has therapeutic effects in musculoskeletal disease. No study has directly tested epitalon in a musculoskeletal disease model. Although epitalon-associated effects on telomere-maintenance pathways have been demonstrated in cellular models, it remains unknown whether these mechanisms translate into improvements in degenerative joint disease or tissue repair. Direct testing in disease-relevant musculoskeletal models should therefore be prioritized.

### 4.2. Melatonin Restoration and Circadian Regulation

Epitalon has also been reported to restore evening melatonin synthesis and normalize circadian cortisol rhythms in senescent rhesus monkeys [20]. This study included six young and six senescent female rhesus monkeys, with three animals in each age/treatment group. Epitalon significantly increased evening melatonin in the senescent animals and was associated with normalization of cortisol rhythmicity [20].

A separate 2005 study of 17 young and 17 old rhesus monkeys found that epitalon administration in aged animals increased nighttime melatonin, decreased basal glucose and insulin, and improved glucose handling [34]. These effects were not observed in younger animals, suggesting that the response may be age-dependent and more pronounced in animals with baseline neuroendocrine dysfunction [34].

Not all experimental findings, however, demonstrate direct stimulation of melatonin secretion. Djeridane et al. evaluated Ala-Glu-Asp-Gly in perifused pineal glands from young and old Wistar rats and found no significant effect on either basal or isoproterenol-stimulated melatonin secretion at the concentrations studied [35]. These findings suggest that epitalon’s reported neuroendocrine effects may depend on experimental context and cannot be attributed simply to direct stimulation of pineal melatonin release.

The potential relevance of these neuroendocrine effects to regenerative medicine is supported indirectly by the broader literature on melatonin and musculoskeletal health. Melatonin has been implicated in bone homeostasis through enhancement of osteoblast activity and osteoclastogenesis via osteoprotegerin (OPG)/receptor activator of nuclear factor κB ligand (RANKL) signaling [40,41]. It has also been reported to protect cartilage by reducing matrix metalloproteinase expression and decreasing oxidative stress mediated by reactive oxygen species [42].

A 2026 systematic review and meta-analysis included 23 randomized trials involving 2028 participants. In chronic musculoskeletal pain, melatonin was not superior to placebo in the primary analysis but performed better than active controls; sensitivity analyses suggested a possible benefit versus placebo [43]. In an observational cohort study, OA patients initiating melatonin had roughly half the risk of subsequent joint replacements compared with those starting benzodiazepines [42].

Whether epitalon offers an advantage over exogenous melatonin remains unknown. Epitalon has been proposed to influence endogenous pineal and circadian function rather than simply providing exogenous melatonin exposure. Epitalon also normalized cortisol rhythms in aged primates, suggesting potentially broader neuroendocrine effects [20,34]. No direct comparison of epitalon with exogenous melatonin has been performed, so any proposed difference between the two remains hypothetical.

Importantly, evidence that melatonin itself influences bone, cartilage, sleep, or pain should not be interpreted as evidence that epitalon produces the same clinical effects. These data establish a mechanistic rationale for investigation rather than therapeutic efficacy of epitalon in musculoskeletal disease.

### 4.3. Antioxidant Defense

Oxidative stress has been implicated in cellular aging and tissue degeneration, and both epithalamin and synthetic epitalon have been investigated for potential antioxidant effects. However, the evidence for the two preparations should be considered separately. Earlier studies of epithalamin reported reductions in lipid peroxidation and increases in endogenous antioxidant activity, including superoxide dismutase (SOD), catalase, and ceruloplasmin. These findings provide historical evidence regarding pineal-derived peptide preparations but cannot be directly attributed to synthetic epitalon [44].

Direct evidence for antioxidant effects of epitalon is more limited. A 2007 study of geroprotective pineal peptides reported antioxidant activity with both epithalamin and epitalon, although much of the broader quantitative antioxidant-enzyme literature derives from epithalamin experiments [36].

More recently, Gatta et al. evaluated synthetic epitalon directly in a high-glucose–injured human retinal pigment epithelial cell model. ARPE-19 cells were treated with epitalon at 20, 40, or 60 ng/mL [37]. High-glucose exposure increased intracellular reactive oxygen species and disrupted antioxidant-associated pathways, whereas epitalon reduced reactive oxygen species (ROS) generation and modulated antioxidant-associated gene expression. Epitalon also restored impaired in vitro wound healing and attenuated hyperglycemia-associated epithelial–mesenchymal transition and fibrotic signaling [37]. These findings support antioxidant-associated activity of synthetic epitalon in a human-derived cellular model, although they do not establish antioxidant efficacy in vivo or in humans.

Oxidative stress is implicated in cartilage degradation, disc degeneration, and impaired wound healing [4]. The cellular findings with epitalon therefore provide a rationale for further investigation, but no study has demonstrated that epitalon improves musculoskeletal disease or tissue repair through an antioxidant mechanism in vivo.

### 4.4. Mitochondrial and Ribosomal Effects in Cellular Aging

Additional in vitro evidence suggests that AEDG may influence cellular features associated with aging. Ivko et al. evaluated AEDG in aging cultures of human pineal-gland cells [38]. The cultures were treated with AEDG at 100 ng/mL during serial subculture, with the 14th passage used as a model of replicative aging. AEDG increased the area of MitoTracker Red mitochondrial staining by approximately 1.5-fold and reduced expression of ribosomal protein L7A by approximately 22% in the aging pineal-cell cultures [38].

The authors interpreted these findings as possible effects on mitochondrial and ribosomal processes during cellular aging. MitoTracker staining, however, is an indirect cellular marker, and the study did not establish systemic mitochondrial effects or clinical benefit. The findings are based on a single in vitro study and have not been independently replicated.

## 5. Preclinical Models

### 5.1. Lifespan and Aging Biomarkers

A 2003 study provided one of the most detailed long-term preclinical evaluations of epitalon using female Swiss-derived SHR mice [21]. Monthly epitalon subcutaneous injections of 1.0 µg per mouse were administered for 5 consecutive days per month, with treatment beginning at 3 months of age and continuing until natural death. Epitalon did not significantly affect mean lifespan, body weight, or food consumption; however, it increased maximum lifespan by 12.3% and extended lifespan among the longest-lived 10% of survivors by 13.3%. The study also found that epitalon slowed age-related loss of estrous function and reduced chromosomal abnormalities in bone marrow cells by 17.1% [21]. The study included 54 animals per group, and mean lifespan was not significantly increased [21]. These findings suggest effects on selected aging-related outcomes but do not demonstrate a generalized increase in lifespan. Selected preclinical studies of epitalon are summarized in Table 2.

**Table 2:** Selected preclinical studies of synthetic epitalon (AEDG).

Study	Model/Setting	n/Experimental Details	Key Findings	Main Limitation	Reference
Anisimov et al. (2003)	Female Swiss-derived SHR mice, in vivo	n = 54/group; SC epitalon 1.0 µg/mouse (~30–40 µg/kg), 5 consecutive days/month from age 3 months until natural death	Mean lifespan unchanged; ↑ maximum lifespan 12.3%; ↑ lifespan of last 10% of survivors 13.3%; ↓ bone-marrow chromosomal aberrations 17.1%	Single research network; aging study rather than formal toxicology; mean lifespan not significantly increased	[21]
Gatta et al. (2025)	Human ARPE-19 retinal epithelial cells under high-glucose injury, in vitro	Epitalon 20, 40, and 60 ng/mL; wound-healing and molecular assays performed across replicate experiments	↓ ROS; improved scratch-wound closure; reduced EMT- and fibrosis-associated signaling	In vitro retinal model; does not establish tissue regeneration in vivo or musculoskeletal efficacy	[37]
Khavinson et al. (2002)	Campbell rats with hereditary retinal degeneration, in vivo	n = 124; 62 epitalon-treated and 62 saline controls; daily parabulbar administration from birth through 72 days	Preserved retinal morphology and prolonged retinal bioelectrical activity compared with saline controls	Disease-specific retinal model; no independent replication	[45]
Chalisova et al. (2015)	Skin explants from young and aged male Wistar rats, ex vivo	800 explants total; 17–20 explants/condition; AEDG 0.001–10 ng/mL; proliferative effects reported principally at 0.05–2.0 ng/mL	↑ growth-zone expansion/proliferation in young and aged skin explants	Ex vivo organotypic model; proliferation does not establish wound healing or regeneration in vivo	[46]
Linkova et al. (2016)	Primary rat skin fibroblasts; replicative-aging model, in vitro	Passage 3 vs. passage 14 cultures; AEDG 20 ng/mL; ≥5 microscopic fields analyzed/condition; biological replicate n not clearly reported	↑ Ki-67 and CD98hc; ↓ caspase-3 and MMP-9 expression in aged cultures	Cellular-marker study; biological replicate n not clearly reported; no in vivo repair endpoint	[47]
Sinjari et al. (2020)	Human periodontal-ligament and gingival mesenchymal stem cells at passage 25, in vitro	AEDG 0.01 µg/mL for 7 days; RT-PCR in 3 independent experiments with duplicate determinations; donor n not clearly reported	↓ p16 and p21 expression in both oral mesenchymal stem-cell types	Senescence-marker study; donor n unclear; no demonstration of improved tissue regeneration or clinical efficacy	[48]
Sibarov et al. (2002)	Male Wistar rats under normal vs. experimental stress, in vivo	n = 24; four groups of 6 rats; intranasal epitalon, four administrations at 12-h intervals	↑ pineal C-Fos under stress; partially reduced stress-associated vascular changes	Small short-term model; effects were stress-dependent and not independently replicated	[22]
Anisimov et al. (2002)	Female FVB/N HER-2/neu transgenic mice, in vivo	90 mice initially randomized; analyzed groups: 28 saline, 25 epitalon, 27 Vilon; SC epitalon 1 µg/mouse, 5 days/month	↓ cumulative mammary tumor number and maximum tumor size; ~3.7-fold ↓ HER-2/neu mRNA	Experimental tumor model; antitumor findings do not establish carcinogenic safety	[49]
Anisimov et al. (2002/2003)	Male LIO rats with DMH-induced intestinal carcinogenesis, in vivo	n = 80, four groups; DMH 21 mg/kg; epitalon 1 µg SC, 5 days/week during different carcinogenesis phases	↓ colon-tumor multiplicity in selected groups and ↓ tumor size with prolonged treatment; follow-up study examined proliferation and apoptosis	Experimental carcinogenesis model; not a formal long-term carcinogenicity assessment	[50,51]

**Abbreviations:** AEDG, Ala-Glu-Asp-Gly; ARPE-19, adult retinal pigment epithelial-19; DMH, 1,2-dimethylhydrazine; EMT, epithelial–mesenchymal transition; HER-2/neu, human epidermal growth factor receptor 2/neu; ROS, reactive oxygen species; RT-PCR, reverse transcription polymerase chain reaction; SC, subcutaneous; ↑, increase; ↓, decrease. **Note:** Sample size is reported where clearly available from the primary study; where a conventional biological n was not clearly reported, the relevant experimental details are provided instead.

### 5.2. Wound Healing and Retinal Models

A 2025 independent in vitro study evaluated the effects of epitalon on wound healing in high glucose-exposed human retinal pigment epithelial cells. Epitalon was tested at 20, 40, and 60 ng/mL and reduced intracellular reactive oxygen species, improved impaired scratch-wound closure, and attenuated epithelial–mesenchymal transition and fibrosis-associated signaling [37].

Additional preclinical retinal evidence has been reported in an animal model of hereditary retinal degeneration. In 124 Campbell rats, including 62 epitalon-treated animals and 62 saline-treated controls, parabulbar epitalon administration was associated with prolonged retinal electrical activity and preservation of retinal morphology compared with saline treatment [45]. This remains a disease-specific retinal model and has not been independently replicated.

Although these findings support further investigation of epitalon in retinal and tissue-repair models, the cellular wound-healing and retinal-dystrophy studies address highly specific experimental systems and should not be extrapolated to musculoskeletal regeneration. No direct musculoskeletal wound-healing study of epitalon has been identified.

Additional preclinical studies have examined the effects of AEDG on skin cells. Chalisova et al. evaluated organotypic skin cultures obtained from young and aged rats. The study included 800 skin explants, with 17–20 explants used for individual experimental conditions, and reported increased growth-zone expansion after AEDG exposure [46]. Linkova et al. subsequently studied replicatively aged rat skin fibroblasts and reported increased Ki-67 and CD98hc expression together with reduced caspase-3 and MMP-9 expression following AEDG treatment [47]. These findings indicate effects on proliferation, apoptosis, and extracellular-matrix remodeling in skin-cell models, but neither study demonstrated improved wound healing or tissue regeneration in vivo [46,47].

A different 2020 study investigated AEDG in cultured human periodontal ligament and gingival mesenchymal stem cells. Cells at passage 25 were treated with AEDG at 0.01 µg/mL for seven days. AEDG reduced p16 and p21 expression in both cell types compared with untreated controls [48]. The experiments suggest an effect on cellular senescence markers in human-derived stem cells. They did not demonstrate enhanced tissue regeneration, improved clinical outcomes, or restoration of stem-cell function in vivo. Independent replication remains necessary.

### 5.3. Neuroendocrine and Stress Protection

In rats exposed to experimental stress, intranasal epitalon increased markers of cellular activity within pineal tissue and reduced stress-related vascular changes within the gland [22]. The study included 24 male Wistar rats divided into four groups of six. The original report found that epitalon modulated pineal activity under stress conditions but not under normal conditions, suggesting a context-dependent effect rather than generalized stimulation of pineal secretion.

Epitalon increased thymocyte proliferative activity under both immune-stimulatory and immune-suppressive stress conditions [52]. Together, these studies suggest that some reported epitalon effects may be more apparent under physiological stress or age-related dysfunction. However, the findings come from small experimental models and have not been independently replicated.

### 5.4. Experimental Carcinogenesis

Epitalon has also been evaluated in several animal carcinogenesis models. In female HER-2/neu transgenic mice, epitalon treatment was associated with reductions in cumulative mammary tumor number, maximum tumor size, and HER-2/neu mRNA expression [49]. Ninety mice were initially randomized among saline, Vilon, and epitalon groups; the analyzed groups contained 28 saline-treated, 27 Vilon-treated, and 25 epitalon-treated animals. Epitalon reduced HER-2/neu mRNA expression approximately 3.7-fold relative to saline-treated controls [49]. The study also reported reduced maximum tumor size and cumulative tumor burden, although tumor incidence and latency were not significantly altered [49]. The original study therefore supports an inhibitory effect within this specific HER-2/neu model rather than a generalized anticancer effect.

In rats exposed to 1,2-dimethylhydrazine (DMH), epitalon reduced colon-tumor multiplicity in selected treatment groups and reduced mean tumor size when administered throughout the experimental period [50]. The study included 80 male LIO rats divided into four groups and compared epitalon administration during different phases of DMH-induced carcinogenesis. A subsequent study using the same experimental model examined effects on tumor-cell proliferation and apoptosis [51].

Importantly, these studies evaluated tumor development and tumor biology rather than carcinogenic safety. These antitumor findings should not be interpreted as evidence that epitalon is noncarcinogenic or that long-term modulation of telomere-maintenance pathways is oncologically safe. The distinction between antitumor activity in selected experimental models and formal carcinogenicity assessment is addressed further in Section 5.5. 

### 5.5. Preclinical Safety

No overt toxicity was described in the cited mouse studies using repeated epitalon administration [21,49,50]. In the lifespan study, food consumption and body weight were not significantly affected [21]. The original lifespan study was nevertheless designed primarily to evaluate aging, lifespan, and spontaneous tumor outcomes rather than formal toxicology.

These observations are therefore insufficient to establish a favorable safety profile. Dedicated dose-ranging toxicology, organ-specific toxicity, genotoxicity, reproductive and developmental toxicity, immunogenicity, and long-term carcinogenicity have not been adequately characterized. The available preclinical literature provides limited tolerability observations but does not establish comprehensive safety.

## 6. Human Evidence

### 6.1. Epithalamin Human Studies

Human studies of epithalamin provide historical context for the development of epitalon but should be interpreted separately because epithalamin is a heterogeneous pineal-derived polypeptide preparation rather than the defined synthetic tetrapeptide AEDG. Accordingly, findings obtained with epithalamin are not considered direct evidence of epitalon efficacy or safety. FDA likewise explicitly distinguishes the two substances.

Khavinson and Morozov reported a clinical assessment of 266 older adults followed for 6–8 years, in whom Thymalin, epithalamin, or both were administered during the first 2–3 years of observation [53]. The authors reported approximately 1.6–1.8-fold lower mortality in the epithalamin-treated group than in controls, together with reductions in several age-associated disease manifestations [53]. Importantly, the interventions included epithalamin and Thymalin, not synthetic epitalon.

A separate 12-year study evaluated epithalamin in 70 elderly patients with coronary disease and accelerated cardiovascular aging. Thirty-six patients received epithalamin and 34 received saline control treatment; the study was described by the authors as randomized. Treatment consisted of five 10 mg intramuscular injections at 3-day intervals every 6 months for 3 years [54]. At 12 years, the investigators reported 28% fewer deaths in the epithalamin group than in controls, approximately two-fold lower cardiovascular mortality, and lower rates of cardiovascular failure and respiratory disease [54]. These findings are notable but derive from a small study conducted within the same broader research network that generated much of the pineal-peptide literature.

A subsequent report by Korkushko et al. described a 15-year follow-up in 79 elderly patients with coronary disease, including 39 who received epithalamin and 40 controls [55]. Because the investigators, patient population, intervention, and follow-up framework closely resemble the earlier study, the two publications may include overlapping participants and should not automatically be treated as independent clinical cohorts [55]. The later report likewise described lower mortality and favorable changes in cardiovascular aging measures.

The 2001 Anisimov et al. report also described antioxidant effects associated with epithalamin in humans and animals [44]. However, this publication is a synthesis of the authors’ prior experimental data rather than a conventional prospective clinical efficacy trial. Its findings therefore provide supportive biological context but should not be interpreted as evidence for synthetic epitalon. Overall, the epithalamin literature suggests possible long-term physiological and mortality associations in elderly populations, but the evidence is methodologically limited and cannot be extrapolated directly to epitalon. The principal human studies of epithalamin are summarized in Table 3.

**Table 3:** Historical human studies of epithalamin.

Study	Population/Setting	n/Intervention Details	Key Findings	Main Limitation	Reference
Khavinson & Morozov (2003)	Adults >60 years, long-term human follow-up	n = 266 total; Thymalin, epithalamin, or both during the first 2–3 years; 6–8-year observation period	Authors reported lower mortality in the epithalamin-treated group (~1.6–1.8-fold vs. control) and fewer age-associated disease manifestations	Heterogeneous treatment groups and limited group-specific methodological detail; historical epithalamin evidence, not synthetic epitalon	[53]
Korkushko et al. (2006)	Elderly patients with coronary disease and accelerated cardiovascular aging; randomized human study	n = 70; 36 epithalamin, 34 control; five 10-mg IM injections at 3-day intervals every 6 months for 3 years; 12-year follow-up	28% fewer deaths reported in treated group; ~2-fold lower cardiovascular mortality; improved exercise tolerance and cardiovascular aging measures	Small study; blinding not clearly described; single research network; long follow-up with limited methodological reporting	[54]
Korkushko et al. (2011)	Elderly coronary patients with accelerated cardiovascular aging; randomized comparative study	n = 79; 39 epithalamin + basic therapy, 40 basic therapy alone; six treatment courses over 3 years; 15-year follow-up	Reported slower cardiovascular aging, preserved physical endurance, normalized melatonin and metabolic rhythms, and lower mortality	Likely overlapping population with the earlier Korkushko report; blinding unclear; should not be treated as independent replication	[55]

**Abbreviations:** IM, intramuscular. **Note:** Epithalamin is a heterogeneous pineal-derived polypeptide preparation and should not be interpreted as direct evidence for synthetic epitalon. The 2006 and 2011 Korkushko reports may include overlapping participants and are therefore not treated as independent replications.

### 6.2. Synthetic Epitalon in Humans

Currently, published human exposure to synthetic epitalon remains limited. The available literature includes small studies evaluating circadian or physiological biomarkers and one older clinical report in retinitis pigmentosa; none evaluates musculoskeletal or pain outcomes. Randomized human exposure studies have been reported, but these studies primarily assessed physiological or circadian biomarkers rather than disease-specific therapeutic outcomes. No adequately powered randomized trial has established clinical efficacy. The available human studies of synthetic epitalon are summarized in Table 4.

**Table 4:** Human studies of synthetic epitalon (AEDG).

Study	Population/Setting	n/Intervention Details	Key Findings	Main Limitation	Reference
Korkushko et al. (2007)	Elderly adults with age-related pineal melatonin dysfunction; human interventional study	Human cohort: 15 epithalamin, 15 epitalon, 10 placebo; 10 young healthy reference subjects. Epitalon IM 0.01 mg/day for 10 days (0.1 mg/course)	Increased nighttime melatonin primarily in participants with reduced baseline pineal function and contributed to normalization of circadian melatonin rhythm	Small groups; physiological biomarker endpoint; no disease-specific efficacy outcome; blinding not clearly described	[23]
Ivko et al. (2020/2021)	Female doctors and nurses aged 40–50 years, primarily night-shift workers; randomized biomarker study	n = 75 total; 35 age-normal reference; 40 with reduced melatonin production randomized 20 placebo/20 AEDG. Sublingual AEDG 0.5 mg/day for 20 days	Urinary 6-sulfatoxymelatonin ↑ ~1.7-fold from baseline; Clock and Csnk1e expression shifted toward reference values; Cry2 expression ↑	Small biomarker study; blinding not specified; no validated sleep or disease-specific clinical endpoint; safety not systematically reported	[24,38,56]
Khavinson et al. (2021)	Women aged 40–59 years, primarily night-shift workers; circadian-gene biomarker study	n = 75 total; 35 reference; 40 with reduced melatonin-forming function randomized to placebo or AEDG. Sublingual AEDG 0.5 mg/day for 20 days	Clock, Cry2, and Csnk1e expression shifted toward values observed in the reference group	Population, intervention, and outcomes closely overlap with [24]; likely related analysis rather than independent replication	[56]
Khavinson et al. (2002)	Patients aged 18–72 years with congenital retinitis pigmentosa; controlled clinical study	n = 162 patients (324 eyes); parabulbar epitalon 5 µg/eye/day for 10 days (50 µg/eye; 100 µg for bilateral course). Control: 46 patients (92 eyes) receiving conventional therapy	Improved retinal electrophysiological measures, visual acuity, and visual fields; positive clinical effect reported in ~90% of treated cases; no treatment-related complications described	Randomization and blinding unclear; conventional therapy rather than matched-placebo control; single-center study without independent replication	[25]

**Abbreviations: AEDG, Ala-Glu-Asp-Gly; IM, intramuscular.** Note: Sample size and intervention details are reported where available from the primary publications. The Ivko and Khavinson night-shift-worker reports appear to involve closely overlapping cohorts and should not be counted as independent clinical replication.

### 6.3. Circadian and Melatonin Studies

Korkushko et al. evaluated epitalon and epithalamin separately in elderly individuals with altered pineal melatonin production [23]. In the human component, elderly participants were assigned to epithalamin, epitalon, or placebo groups. Epitalon was administered intramuscularly at 0.01 mg daily for 10 days, whereas epithalamin was administered using a substantially different regimen. The authors reported increased nighttime melatonin primarily in participants with reduced baseline pineal function and partial normalization of the circadian melatonin rhythm following epitalon treatment [23].

These findings should be interpreted as physiological biomarker effects rather than evidence of clinical efficacy. The study did not establish improvement in a disease-specific clinical outcome, and the epitalon and epithalamin treatment groups should not be pooled or discussed interchangeably.

Ivko et al. subsequently evaluated AEDG in middle-aged female night-shift workers [24]. Seventy-five participants aged 40–50 years were initially evaluated; 35 with age-appropriate urinary 6-sulfatoxymelatonin values served as a reference group, whereas 40 with reduced melatonin production were randomly divided into placebo and AEDG groups. AEDG was administered sublingually for 20 days at approximately 0.5 mg/day. Treatment was associated with an approximately 1.7-fold increase in urinary 6-sulfatoxymelatonin, a reduction in Clock and Csnk1e expressions toward reference values, and an increase in Cry2 expression [24]. The full study reports these same biomarker changes. However, the study did not evaluate validated sleep outcomes or another disease-specific therapeutic endpoint, blinding was not specified, and safety data were not systematically reported. Therefore, the study supports biological activity at the level of circadian biomarkers but does not establish clinical benefit.

A related 2021 publication by Khavinson, Linkova, and Ivko examined Clock, Cry2, and Csnk1e expression in a highly similar population of 75 middle-aged female night-shift workers aged 40–59 years, including 40 participants with reduced melatonin-forming function who were randomly divided between placebo and sublingual AEDG groups [56]. Because the population, intervention, and reported outcomes closely overlap with those of the Ivko study, these reports appear to represent closely related analyses and should not be interpreted as independent clinical replication unless cohort independence can be demonstrated [24]. FDA likewise discussed the two publications separately but identified both as part of the limited direct human epitalon evidence base [18].

### 6.4. Retinitis Pigmentosa

A clinical study evaluated epitalon in 162 patients (324 eyes) aged 18–72 years with congenital retinitis pigmentosa [25]. Epitalon was administered by parabulbar injection at 5.0 µg per eye once daily for 10 days, corresponding to 50 µg per eye and 100 µg for bilateral treatment. The control group included 46 patients, representing 92 eyes, who received conventional therapy [25].

Treatment was associated with improvements in retinal electrophysiological measures, visual acuity, and visual fields. The authors reported a positive clinical effect in approximately 90% of treated cases [25]. PubMed likewise describes improvement in retinal functional measures and a positive clinical effect in about 90% of cases. No worsening of clinical condition was reported during treatment, and no treatment-related complications or adverse effects were described [25].

However, randomization and blinding were not clearly described, and the control group received conventional therapy rather than a matched placebo. The absence of a clearly described allocation procedure, masking, and placebo control substantially limits causal interpretation. These findings provide a preliminary clinical signal in retinitis pigmentosa but are insufficient to establish therapeutic efficacy.

### 6.5. Overall Interpretation of Human Epitalon Evidence

Human evidence for synthetic epitalon remains limited to small physiological or biomarker studies and one older controlled retinal study with important methodological limitations. Randomized placebo-controlled exposure has been reported, but no adequately powered randomized trial has demonstrated clinical efficacy for aging, longevity, sleep disorders, musculoskeletal disease, pain, or another regenerative indication.

Human pharmacokinetics, dose-ranging studies, and dedicated prospective safety trials also remain unavailable. The distinction between biological activity and therapeutic benefit is especially important in this evidence base. Changes in urinary melatonin metabolites or circadian-gene expression demonstrate physiological effects but do not by themselves establish improvement in clinically meaningful outcomes.

Taken together, the human literature supports evidence of biological activity after epitalon exposure, but therapeutic efficacy remains unproven. Historical epithalamin findings should remain separate because the two preparations are chemically distinct, and the existing epitalon studies are too small and methodologically limited to support conclusions regarding longevity, regenerative medicine, or routine clinical use.

## 7. Safety, Regulatory, and Ethical Considerations

### 7.1. Safety Evidence and Current Limitations

The safety of epitalon remains insufficiently characterized. Although overt adverse effects were not reported in several published animal studies, these experiments were primarily designed to evaluate lifespan, physiological, or tumor-related outcomes rather than formal toxicology [21,49,50]. The absence of reported toxicity in these studies therefore cannot establish a favorable safety profile.

Human safety evidence is particularly limited. Published human safety data for epitalon are minimal and generally derive from small or methodologically limited cohorts; none of the available studies were designed as dedicated prospective safety studies. In the sublingual AEDG study of night-shift workers, adverse-event data were not reported systematically [24]. The 2007 study of elderly individuals reported no apparent adverse effects with epitalon or epithalamin, but detailed methods for adverse-event ascertainment were limited [23]. No adequate human dose-escalation, pharmacokinetic, pharmacodynamic, or long-term prospective safety studies have been identified.

FDA’s 2026 evaluation similarly found that the available data were insufficient to establish the safety of epitalon-related bulk drug substances in humans, particularly when administered subcutaneously [18]. FDA specifically stated that it had not identified clinical safety data sufficient to determine whether epitalon free base or epitalon acetate would cause harm in humans. Important unresolved areas include dose-dependent toxicity, organ toxicity, immunogenicity, genotoxicity, reproductive and developmental toxicity, and long-term carcinogenicity. Accordingly, the lack of reported adverse events should not be interpreted as evidence of safety in the absence of adequate safety characterization.

### 7.2. Telomerase Activation and Oncologic Considerations

Telomerase activation represents both one of epitalon’s principal proposed mechanisms and an important unresolved safety consideration. Telomerase promotes maintenance of telomere length and may delay replicative senescence; however, telomere-maintenance pathways also play a central role in cellular immortalization and cancer biology.

The corrected 2025 cellular study reported telomere elongation in both normal and malignant human cell models, with differential effects on telomerase and ALT pathways across cell types [11]. These findings do not demonstrate that epitalon causes cancer, but they indicate that epitalon can influence telomere-maintenance pathways in malignant as well as nonmalignant cells. The long-term biological consequences of these effects remain unknown.

Animal studies have reported antitumor activity in HER-2/neu transgenic mice and chemically induced colon carcinogenesis models [49,50,51]. However, these findings should not be interpreted as demonstrating carcinogenic safety. An intervention may reduce tumor growth in selected experimental models while still having unresolved effects on tumor initiation, dormant malignant cells, or long-term telomere-dependent oncogenic processes. Formal carcinogenicity studies designed specifically to address these questions have not been reported.

A 2026 study also evaluated epitalon in experimental models of chemotherapy-resistant natural killer/T-cell lymphoma. Epitalon was used as an inhibitor of a downstream senescence-associated regulatory pathway, and the combination of epitalon and chaetocin produced greater antitumor effects than either single agent or control treatment in the mouse model. The combination also reduced senescence-associated markers and partially reversed the immunosuppressive, chemoresistant phenotype [57]. These findings remain preclinical and do not establish anticancer efficacy or long-term oncological safety in humans.

FDA’s 2026 scientific review similarly identified telomerase activation and telomere elongation as a potential mechanistic carcinogenic concern and concluded that available nonclinical studies were too limited in scope and duration to characterize this risk adequately [18]. Thus, the existing evidence neither demonstrates that epitalon is carcinogenic nor establishes long-term oncological safety. This uncertainty warrants dedicated genotoxicity, carcinogenicity, and long-duration studies before chronic human exposure can be adequately evaluated.

### 7.3. Immunogenicity, Aggregation, and Product Quality

Product-related safety concerns include peptide aggregation, synthesis-related impurities, microbiological quality, and immunogenicity. FDA emphasized that these risks may be particularly relevant to injectable preparations and that peptide stability and immunogenicity depend heavily on manufacturing and finished-product quality [18]. FDA specifically identified insufficient information regarding peptide-related impurities and aggregation and noted that these factors may increase immunogenic risk with certain routes of administration [18].

These concerns are particularly relevant to products obtained outside regulated pharmaceutical manufacturing pathways. The chemical identity of AEDG does not by itself guarantee equivalent purity, sterility, concentration, or stability among commercially marketed preparations. Therefore, adverse-event experience with one formulation cannot be generalized to other compounded or gray-market products.

### 7.4. Regulatory Status

Epitalon is not an FDA-approved drug in the United States. In July 2026, the FDA Pharmacy Compounding Advisory Committee (PCAC) considered epitalon free base and epitalon acetate for potential inclusion on Section 503A Bulk Drug Substances List for the nominated use of insomnia [18]. FDA’s meeting materials confirm that the free-base and acetate forms were evaluated separately and that insomnia was the use under review.

FDA staff concluded that the balance of available evidence weighed against inclusion of either epitalon free base or epitalon acetate on the 503A Bulks List. FDA cited incomplete physical and chemical characterization, insufficient human safety data, potential immunogenicity concerns, limited information on historical compounding use, and insufficient evidence of effectiveness for insomnia [18].

The advisory committee subsequently recommended inclusion of epitalon free base and epitalon acetate, contrary to the FDA staff recommendation. That committee recommendation is advisory and nonbinding and does not constitute FDA approval, establish clinical efficacy or safety, or itself place epitalon on the 503A Bulks List. At the time of writing, epitalon is not an FDA-approved treatment for insomnia, longevity, musculoskeletal disease, pain, or any other indication.

### 7.5. Ethical and Clinical Considerations

Public interest in peptide-based therapies has increased substantially, while the regulatory landscape surrounding their use remains unsettled. A 2026 JAMA article noted substantial regulatory uncertainty surrounding unapproved peptide products [10]. This environment creates challenges for clinicians because public interest and commercial availability may substantially outpace the quality of clinical evidence.

In discussions with patients, epitalon should therefore be described as an investigational peptide that is not FDA-approved and has mechanistic and preclinical evidence but insufficient clinical evidence to support routine therapeutic use. Clinicians should distinguish clearly among three separate concepts: biological activity, clinical efficacy, and regulatory availability. Evidence that epitalon alters telomerase activity or circadian biomarkers does not establish clinical benefit, and potential compounding eligibility does not constitute FDA approval. Given that much of the epitalon literature originates from a limited number of investigators, independent replication, standardized product characterization, formal toxicology, pharmacokinetic studies, and prospective human safety trials remain necessary before clinical translation can be supported.

## 8. Translational Considerations and Future Research

**Figure 2:**
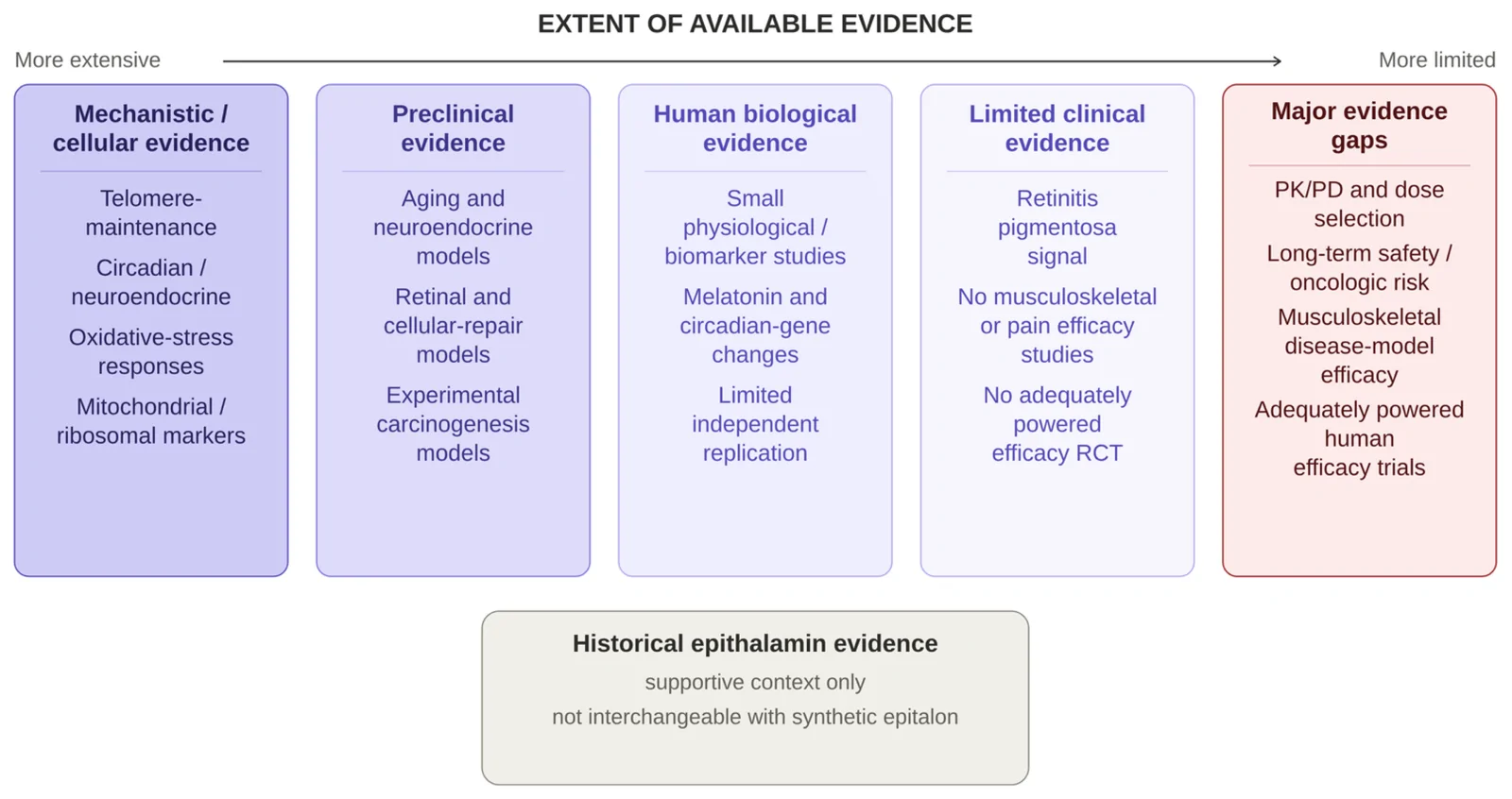
Current evidence for synthetic epitalon. Mechanistic and preclinical evidence is more developed than human therapeutic evidence. Human studies predominantly evaluate physiological or biomarker outcomes, and no adequately powered randomized trial has established efficacy for regenerative, musculoskeletal, pain, or longevity indications. Historical epithalamin evidence is presented as supportive context only and should not be considered directly interchangeable with evidence for synthetic epitalon. Abbreviations: AEDG, Ala-Glu-Asp-Gly; PK, pharmacokinetics; PD, pharmacodynamics; RCT, randomized controlled trial.

The current epitalon literature demonstrates biological activity across several experimental systems but does not yet provide a sufficient foundation for efficacy-focused trials in regenerative or pain medicine. Existing evidence includes cellular telomerase studies, animal neuroendocrine and longevity models, limited human physiological or biomarker studies, and small historical clinical reports, but no adequately powered randomized trial has established therapeutic efficacy in regenerative or musculoskeletal disease [11,20,21,23,24]. No direct epitalon study evaluating therapeutic efficacy in osteoarthritis, intervertebral-disc degeneration, tendinopathy, or another musculoskeletal disease model was identified in our literature search. The current evidence landscape for synthetic epitalon, including the distinction between mechanistic, preclinical, and human evidence, is summarized in Figure 2. Future development should therefore follow a staged translational pathway addressing compound characterization, pharmacology, safety, and indication-specific efficacy before broader clinical investigation.

First, a reproducible, well-characterized pharmaceutical formulation should be established, with assessment of identity, purity, stability, aggregation, and relevant peptide-related impurities. FDA’s 2026 evaluation identified important gaps in the physical and chemical characterization of both epitalon free base and epitalon acetate, including limited information regarding impurities and aggregation [18]. These issues should be resolved before pharmacological and safety findings from a particular formulation can be interpreted or generalized.

Formal pharmacokinetic and pharmacodynamic characterization is also needed. FDA reported that no pharmacokinetic data were identified for either epitalon free base or epitalon acetate [18]. Future studies should characterize absorption, distribution, metabolism, elimination, systemic exposure, and relationships between dose and relevant pharmacodynamic biomarkers. Because substantially different administration routes have been used across the existing literature, route-specific bioavailability and exposure should also be established rather than assuming comparable systemic delivery across formulations or routes. These data are necessary for rational dose selection and for meaningful comparison among future studies.

Second, dedicated nonclinical safety studies should evaluate dose–response relationships, repeated-dose toxicity, immunogenicity, genotoxicity and carcinogenic risk, with reproductive or developmental assessment as appropriate to the intended clinical use. FDA found the available nonclinical safety evidence insufficient to characterize important risks and specifically highlighted uncertainties surrounding immunogenicity and long-term carcinogenic potential [18].

Given epitalon’s reported effects on telomere-maintenance pathways, oncologic safety warrants particular attention. The corrected 2025 study reported that epitalon can alter telomere biology in both normal and malignant human cell models, through telomerase-associated and ALT-associated responses that differ by cell type [11]. Existing antitumor findings in selected animal models and the more recent natural killer/T-cell lymphoma study are biologically relevant but cannot substitute for formal assessment of long-term oncologic risk [49,50,51,57].

Third, disease-relevant preclinical testing is required before musculoskeletal applications can reasonably progress toward clinical efficacy studies. Appropriate experimental models could include osteoarthritis, intervertebral-disc degeneration, tendon injury, or other degenerative conditions in which structural, inflammatory, functional, and mechanistic outcomes can be assessed together. Associations between telomere shortening, oxidative stress, melatonin biology, and musculoskeletal disease do not establish that epitalon itself produces therapeutic effects in these conditions [4,16,17,40,41,42,43]. These conditions should therefore be viewed as potential areas for future investigation rather than established or evidence-supported indications for epitalon.

Such studies should determine whether the cellular effects reported with epitalon translate into meaningful changes in disease-relevant tissue outcomes [11]. Mechanistic biomarker changes alone should not be considered sufficient evidence of regeneration or disease modification.

If a standardized formulation subsequently demonstrates an acceptable nonclinical safety profile and interpretable pharmacokinetic and pharmacodynamic characteristics, early-phase human studies should focus first on safety, tolerability, dose selection, and exposure-response relationships rather than therapeutic efficacy. Current human epitalon studies demonstrate biological activity through melatonin and circadian biomarkers, but these findings do not establish clinical benefit [23,24]. Only after these foundational data are available should adequately powered, indication-specific randomized trials evaluate clinically meaningful outcomes such as pain, physical function, structural disease progression, or other validated disease-specific endpoints.

Independent replication should remain a central priority throughout this process. Much of the historical epitalon literature originates from a limited number of collaborating investigators, while independent confirmation remains sparse. The 2025 Al-Dulaimi et al. study provides an important source of evidence from investigators outside the historical epitalon research network, but one independent study is insufficient to establish reproducibility across mechanisms or experimental systems [11]. Broader independent replication across cellular models, animal studies, and human investigations remains necessary.

## 9. Conclusions

Epitalon is a synthetic tetrapeptide that has been investigated for effects on several biological processes associated with aging, including telomere maintenance, circadian regulation, and oxidative stress. The strongest direct mechanistic evidence involves modulation of telomere-maintenance pathways in human cell models. The corrected 2025 study reported differential effects on telomere-maintenance pathways in normal and malignant human cell models [11]. These findings provide important evidence from investigators outside the historical epitalon research network, but broader independent replication remains limited.

Preclinical studies have also reported neuroendocrine effects in aged primates, selected aging-related outcomes in mice, and antioxidant-associated and tissue-protective effects in cellular models [20,21,37]. These findings remain predominantly mechanistic or preclinical and should not be interpreted as evidence of established regenerative, musculoskeletal, or longevity benefits in humans.

Human evidence for synthetic epitalon remains limited. Small studies have demonstrated changes in melatonin-related and circadian biomarkers, while an older controlled study in retinitis pigmentosa reported a preliminary clinical signal but had important methodological limitations, including unclear randomization and blinding [23,24,25]. No adequately powered randomized trial has established therapeutic efficacy. Importantly, historical findings obtained with epithalamin should not be extrapolated directly to epitalon because the two preparations are chemically distinct.

Important pharmacological and safety gaps also remain. FDA’s 2026 evaluation identified no pharmacokinetic data for epitalon free base or epitalon acetate, and adequate human dose-ranging and long-term prospective safety studies remain unavailable [18]. The safety profile of epitalon therefore remains insufficiently characterized.

Its effects on telomerase and telomere-maintenance pathways warrant particular attention because the long-term oncologic consequences of these mechanisms remain unknown [11]. Although selected animal studies reported reductions in tumor burden or specific tumor-related endpoints, these findings do not establish long-term carcinogenic safety [49,50,51]. FDA’s 2026 evaluation similarly identified unresolved concerns regarding long-term carcinogenic potential, immunogenicity, aggregation, peptide-related impurities, and overall human safety [18].

For regenerative and pain medicine, epitalon should currently be regarded as biologically active but clinically unproven. No direct epitalon study establishing therapeutic efficacy in a musculoskeletal disease model was identified in our literature search. Associations between telomere biology, melatonin, oxidative stress, and musculoskeletal degeneration provide a rationale for further investigation but are not evidence that epitalon improves these conditions.

Future development should proceed in a staged manner, beginning with standardized pharmaceutical characterization, pharmacokinetic and pharmacodynamic studies, formal nonclinical safety assessment, and independent replication. Disease-specific preclinical testing should precede early-phase human safety and dose-selection studies, and efficacy-focused randomized trials should be considered only after these foundational gaps have been addressed.

## Data Availability

No new data were created or analyzed in this study. Data sharing is not applicable to this article.

## Supplementary Materials

The following supporting information can be downloaded at: https://www.mdpi.com/article/10.3390/ijms27198922/s1.

## Abbreviations

ALT	alternative lengthening of telomeres.
BMAC	bone marrow aspirate concentrate.
BPC-157	Body Protection Compound-157.
EMT	epithelial–mesenchymal transition.
FDA	U.S. Food and Drug Administration.
hTERT	human telomerase reverse transcriptase.
IND	Investigational New Drug.
MMP	matrix metalloproteinase.
OA	osteoarthritis.
PCAC	Pharmacy Compounding Advisory Committee.
PK	pharmacokinetic.
PRP	platelet-rich plasma.
RCT	randomized controlled trial.
ROS	reactive oxygen species.
RPE	retinal pigment epithelium.
SASP	senescence-associated secretory phenotype.
SOD	superoxide dismutase.
SPPS	solid-phase peptide synthesis.
