# Supplementary material for: Epitalon and Epithalamin: A Narrative Review of Mechanistic, Preclinical, and Clinical Evidence
Source: Int J Mol Sci. 2026 Oct 8;27(19):8922. doi: 10.3390/ijms27198922 (PMC13664938; doi:10.3390/ijms27198922)
Supplement: Supplementary file 1 [file ijms-27-08922-s001.zip › ijms-4557144-supplementary.pdf]

Table S1. Literature search strategies and retrieval details.

Database	Exact search query	Retrieval and screening
PubMed	("epitalon"[Title/Abstract] OR "epithalon"[Title/Abstract] OR "epithalone"[Title/Abstract] OR "AEDG"[Title/Abstract] OR "Ala-Glu-Asp-Gly"[Title/Abstract] OR "epithalamin"[Title/Abstract] OR "epithalamine"[Title/Abstract])	233 records retrieved in the September 22 update. Title/Abstract fields; no date or language restrictions.
Google Scholar	epitalon OR epithalon OR epithalone	First five results pages examined.<br>Relevance order; no date or language restrictions.
Google Scholar	epithalamin OR epithalamine	First five results pages examined.<br>Relevance order; no date or language restrictions.
Google Scholar	"AEDG" peptide senescence	First five results pages examined.<br>Relevance order; no date or language restrictions.

Note*:* Initial searches were conducted on August 26, 2026, and updated on September 22, 2026. Google Scholar screening was limited to the first five results pages per query as a supplementary search within the narrative scope of this review. Result-page entries may overlap across searches; no combined unique-record count is reported. The PubMed total refers to records retrieved, not studies included. The exact number of Google Scholar entries examined and the status of citation-only results were not independently documented.
